# Preliminary Examination of an Innovative mHealth-Based Dietary Fiber Intervention to Improve Outcomes in Young Adults With Prediabetes: Protocol for a Single-Arm Feasibility Study

**DOI:** 10.2196/97873

**Published:** 2026-09-23

**Authors:** Ashlea C Braun, Sarah Corcoran, Dorsa Hosseininasab, Marah Johnson, Fetemeh Kochackpour, Emily T Hébert, T Kent Teague, Martina J Jelley, Summer G Frank-Pearce, Rachel Liebe, Laura J Chalmers

**Affiliations:** 1 TSET (Tobacco Settlement Endowment Trust) Health Promotion Research Center, OU (University of Oklahoma) Health Stephenson Cancer Center, 655 Research Parkway Suite 400, Oklahoma City, OK, 73104, United States, 1 405-271-6872; 2 Health Promotion Sciences, Hudson College of Public Health, University of Oklahoma Health Campus, Oklahoma City, OK, United States; 3 Health and Exercise Science Department, University of Oklahoma, Norman, OK, United States; 4 Department of Family and Preventive Medicine, University of Oklahoma Health Campus, Oklahoma City, OK, United States; 5 Department of Surgery, University of Oklahoma School of Community Medicine, Tulsa, OK, United States; 6 Department of Internal Medicine, University of Oklahoma School of Community Medicine, Tulsa, OK, United States; 7 Biostatistics & Epidemiology, Hudson College of Public Health, University of Oklahoma Health Campus, Oklahoma City, OK, United States; 8 Department of Nutritional Sciences, Oklahoma State University, Stillwater, OK, United States; 9 Diabetes and Endocrinology, University of Oklahoma School of Community Medicine, Tulsa, OK, United States

**Keywords:** prediabetic state, dietary fiber, biofeedback, telemedicine, psychology

## Abstract

**Background:**

One in 4 young adults has prediabetes, and improving diet is a key step to lowering the risk of diabetes. Existing standard-of-care diet approaches show short-term efficacy but lack long-term effectiveness, including in young adults. Greater fiber intake is associated with a reduced risk of diabetes; however, fiber is not well targeted using existing interventions. Many young adults may be reluctant to consume fiber given the coexistence with digestible carbohydrates and concerns over gastrointestinal effects. Targeting these factors more explicitly may improve uptake and lower diabetes risk, and doing so via a mobile health (mHealth) intervention may be particularly responsive to young adult demands.

**Objective:**

This study aims to establish and preliminarily test the feasibility of a highly innovative and scalable mHealth-based intervention to improve fiber intake among young adults with prediabetes.

**Methods:**

This single-arm feasibility study includes 2 phases to preliminarily test an mHealth-based fiber intervention (phase 1) and elucidate factors that impact sustained behavior change and fiber intake after intervention end using ecological momentary assessment (EMA; phase 2). The intervention is 3 months in length and features daily EMAs with responsive text–based coping messages to target key predictors of fiber intake. EMAs will also be paired with brief (ie, <1 minute) educational videos on fiber delivered once per day. Participants will also receive weekly home-delivered high-fiber food packages and wear a continuous glucose monitor at 2 time points during the intervention as a form of biofeedback. At the baseline and postintervention time points, fasting blood glucose, hemoglobin A_1c_, and insulin resistance (Homeostatic Model Assessment of Insulin Resistance) will be assessed. After the postintervention time point, participants will continue with daily EMAs to assess factors associated with sustained fiber intake. Diet will be assessed using Automated Self-Administered 24-hour dietary recalls at the baseline and postintervention time point and after the phase 2 EMA.

**Results:**

This study was initiated in July 2025. As of March 2026, the mHealth intervention content is being built, with app completion and trial enrollment both slated to begin in April 2026.

**Conclusions:**

The results of this study will provide pivotal evidence on the utility of a fiber-focused intervention delivered via mHealth with biofeedback and home-delivered foods to lower the risk of diabetes in young adults with prediabetes.

## Introduction

One in 4 young adults has prediabetes [1]. Diet is instrumental in diabetes prevention [2]. Existing standard-of-care diet approaches (eg, Diabetes Prevention Program; DPP) promote a negative energy balance via multiple behaviors (eg, decreased fat intake, decreased processed food intake, counting calories, etc). This negative energy balance enables weight loss, which staves off diabetes development. While the DPP shows short-term efficacy for decreasing the incidence of diabetes, in the long term, DPP participants are nearly identical to controls as weight gain is still observed long after intervention completion [3]. These null effects are particularly pronounced in young adults [3].

Key limitations of the DPP and similar complex interventions are the incompatibility of intervention mechanisms with daily life [4]. That is, maintaining a negative energy balance without ongoing, intensive intervention is exceedingly difficult. End users report a desire for alternative interventions that promote behaviors that are easier to maintain without intervention, including via the use of clear messaging [4-6]. When interventions are more easily maintained, adherence improves, ultimately improving behavior adoption and long-term disease risk reduction [4].

Interventions focused singularly on improving fiber intake offer a simplified approach to modifying behavior [5,7,8]. Fiber interventions perform similarly and, in some ways, are superior to complex alternatives [5,7,9]. In addition, regular intake of fiber—a nondigestible carbohydrate found only in plant foods—is associated with significantly lower diabetes risk [10-12]. Higher intake of fiber from grains (eg, wheat) is associated with 10% to 11% lower diabetes risk [2,12], higher intake of legumes is associated with 35% lower diabetes risk [13], higher intake of nuts is associated with 35% to 43% lower diabetes risk [12], and higher intake of vegetables is associated with 4% lower diabetes risk [2]. Fiber improves glycemic control via multiple mechanisms, including delayed gastric emptying, increased satiety, decreased glucose absorption, decreased inflammation, and increased uptake of glucose into muscle tissue [14-16]. Interventions focused on fiber can also address the fact that fiber intake has been insufficient for decades, with adults eating approximately 16 grams per day, nearly half the recommendation [17-19]. Fiber is mentioned in interventions such as the DPP but only briefly. Thus, post-DPP fiber intake does not improve to levels that are meaningful (17.8 g fiber per 2000 kcal vs the recommended amount of >28 g per 2000 kcal [17,18,20]). Some of the known effects of fiber (eg, delayed gastric emptying and microbiota modulation) can also lead to gastrointestinal (GI) side effects that may be perceived as uncomfortable, discouraging intake over time [21,22]. These are not explicitly targeted as part of many standard intervention approaches. More directly targeting these, including the use of anticipatory guidance and coping messages, may improve uptake of fiber recommendations.

Interventions that place a singular emphasis on fiber may improve outcomes given the simplified intervention approach and distinct health effects of fiber. Research has shown that fiber intake is a top predictor of long-term success following interventions [23]. Ma et al [5] found that an in-person intervention with a “single-component” goal of increasing fiber intake significantly improved insulin resistance compared to a “multicomponent” goal of adhering to all American Heart Association diet recommendations. The intervention included a combination of group classes and counseling over 12 months. Intervention engagement was only 56%, so there was room for improvement in intervention delivery; however, the content appears promising [5]. Mobile health (mHealth) interventions hold the potential to increase engagement due to the ubiquity of mobile phones [4]. mHealth-based DPP versions show high retention (>80%) [24,25], whereas young adult–specific mHealth interventions improve other markers of diet quality (eg, vegetable intake) [26]. Few, if any, mHealth interventions have been used to target fiber intake. mHealth interventions may also address common reluctance to procure unfamiliar foods by augmenting with home delivery of high-fiber foods [27]. Tester and Leak [9] found that sending high-fiber foods to adults significantly improved fasting glucose and measures of insulin resistance.

There is also a desire for mHealth interventions that include biofeedback, or “direct feedback about the body using external monitoring devices” [4,28]. Continuous glucose monitors (CGMs) are used in diabetes treatment but less as a diet-focused behavior change tool in prediabetes. CGMs have been used as a behavior change tool to promote exercise in adults with diabetes or prediabetes, improving goal setting, exercise, and waist circumference (*P*<.05) [29]. Use of CGMs may also address emerging concerns regarding fiber’s glycemic effects. As fiber is a carbohydrate and is often present with other digestible carbohydrates such as starch, there may be fear that fiber—or foods that contain fiber—should be avoided [30]. In pursuit of simplified interventions, other approaches (eg, carnivore diets) that eliminate carbohydrates and plants in total have grown popular. This has led to a potential collateral demonization of fiber [31,32]. This is unfortunate given that fiber from whole foods—even whole foods high in carbohydrates, such as cereal grains and legumes—are associated with lower diabetes risk [12,33].

Short-term changes in behavior are common in diet interventions; however, achieving changes that are sustained after the intervention remains elusive [3]. Ecological momentary assessments (EMAs) are often used to assess dynamic factors that contribute to behavior [34] and can be used to elucidate sustained intervention response [35]. Monitoring behavior after an intervention is common in tobacco- and alcohol-focused EMA studies [35]. However, diet-focused EMA studies more commonly assess diet itself (eg, foods eaten) or nonfood factors (eg, stress) during the intervention [36-40]. For example, Goldstein et al [40] used EMA during a 12-month weight loss intervention to assess behavior that influenced response during the intervention (eg, feeling deprived predicted eating unintended foods; *P*<.05) but not after the intervention. Moreover, little work exists using EMA to measure response to fiber ingestion, which could fill key knowledge gaps regarding how fiber affects appetite and behavior. In a 3-year weight maintenance intervention, Zhu et al [41] found that fiber intake was associated with improved satiety but increased desire to eat; however, this study assessed these factors at 5 separate weeks throughout the 3-year period, offering little insight into dynamic effects. Therefore, the objective of this study is to establish and preliminarily test the feasibility of a highly innovative and scalable mHealth-based intervention to improve dietary fiber intake among young adults with prediabetes.

## Methods

### Study Design

**Figure 1.**
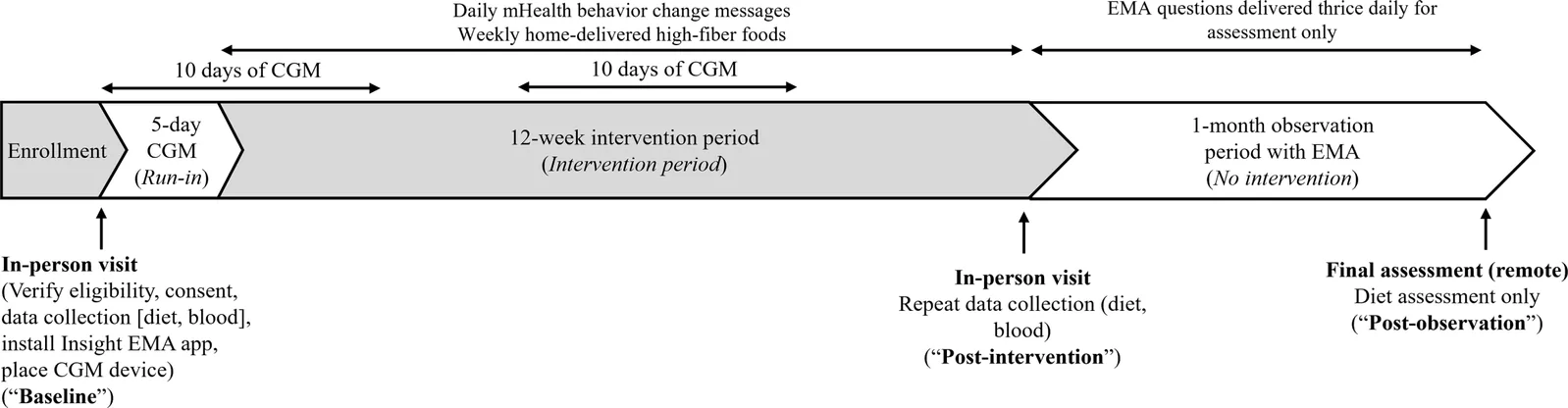
Study design and schematic. CGM: continuous glucose monitoring; EMA: ecological momentary assessment; mHealth: mobile health.

On the basis of our primary study outcome (feasibility), we will conduct a 2-phase single-arm study [42,43] to preliminarily test our mHealth-based fiber intervention in up to 80 young adults with prediabetes (phase 1) and elucidate factors that impact sustained behavior change and fiber intake after intervention end using EMAs (phase 2) (Figure 1).

### Theoretical Framework

The theoretical framework for this intervention is a combination of self-determination theory (SDT), social cognitive theory (SCT), and the biopsychosocial model [44-46]. In brief, the intervention taps into SDT given that it honors individual autonomy (eg, allows for flexibility in the remainder of the diet outside of fiber), promotes competence (eg, targets knowledge), and promotes relatedness (eg, use of interactive mHealth content). It also taps into SCT given that it alters cognitive processes and integrates suggestions from previous participants (observational learning). Finally, it intentionally integrates elements of coping to address the expected yet physiologically normal GI responses that can occur with increased fiber ingestion [21,22]. These messages are informed by both cognitive behavioral therapy and acceptance and commitment therapy, featuring elements of anticipatory coping (eg, preparing individuals for potential symptoms), behavioral coping strategies (eg, gradual fiber increases, hydration, and symptom monitoring), and elements of cognitive reframing [47].

### Participants

We will recruit young adults aged 18 to 39 years who have prediabetes [48]. Classification of prediabetes is based on having a hemoglobin A_1c_ (HbA_1c_) level between 5.7% and 6.4% per American Diabetes Association guidelines [49]. Other inclusion criteria are residing in or near Tulsa, Oklahoma (where in-person data collection will take place), or nearby and being willing to travel for visits; ability to access and/or use a smartphone; and proficiency in the English language. Exclusion criteria are a suspected eating disorder based on the 26-item Eating Attitudes Test (which was reproduced with permission [50]), current use of a glucagon-like peptide-1 receptor agonist, food allergies or intolerances that preclude engagement, self-reported diagnosis of irritable bowel syndrome or inflammatory bowel disease, self-reported pregnancy or breastfeeding, or travel plans that limit intervention completion.

### Recruitment

Participants will be recruited via community events; email distributions to students, faculty, and staff at the University of Oklahoma; directly via the medical record system of the University of Oklahoma Health Campus (MyChart); social media advertisements; provision of flyers at university health screenings; and direct communication and distribution of flyers in the clinics within internal medicine at the University of Oklahoma Health Campus in Tulsa. Prospective participants can provide documentation of an HbA_1c_ level within the prediabetes range from the previous 3 months or complete the Centers for Disease Control and Prevention Prediabetes Risk Test as part of the study screening. If prospective participants are otherwise eligible and appear to be at risk of prediabetes per the Prediabetes Risk Test, they will be invited to attend an in-person recruitment visit wherein they can complete the informed consent process, followed by a screening HbA_1c_ test. If their HbA_1c_ level is within the prediabetes range, they will be eligible for enrollment in the study. If their HbA_1c_ level is not within the prediabetes range, their participation will be considered complete, and they will be deemed ineligible.

### Ethical Considerations

University of Oklahoma Health Campus Institutional Review Board approval was obtained on August 18, 2025. Written informed consent will be obtained from all individuals in person prior to any study-related screening of HbA_1c_ and (if eligible) study participation. During the informed consent process, participants will be informed of potential risks, including those that are expected to occur with increases in fiber intake (eg, GI discomfort). Participants will receive compensation in the form of gift cards for completion of initial HbA_1c_ screening (US $15), US $50 for the follow-up visit if applicable, and up to US $168 for EMAs across both phases (up to US $84 for phase 1 and up to US $84 for phase 2). Rates of EMA compensation will be based on the rate of EMA completion: participants who complete 50% (42/84) to 73.8% (62/84) of EMAs will receive a US $42 gift card, those who complete 75% (63/84) to 89.3% (75/84) of EMAs will receive a US $63 gift card, and those who complete 90.2% (>75/84) or more will receive a US $84 gift card [51].

Data will be primarily collected in REDCap (Research Electronic Data Capture; Vanderbilt University). EMA data will be collected in the University of Oklahoma Health Campus OU Health Stephenson Cancer Center mHealth Shared Resource (Insight). No protected health information will be stored or transmitted via Insight. CGM data will be accessed via participant Clarity app or Dexcom accounts for data extraction. All data will be ultimately coded and saved on OU Health specific platforms and/or REDCap. EMA and CGM data will be monitored for the corresponding intervention periods. If blood glucose levels remain abnormally elevated, consultation with the remainder of the team (eg, MJJ and LJC) will be made, and appropriate action will be taken. Participants will also be given guidance on actions to take if blood glucose remains high. In addition to study team contact information, participants will be provided with an individualized REDCap link to report any adverse events, which will be promptly reviewed by the research team and reported according to (1) whether they were related to the study, (2) whether they were anticipated, and (3) level of severity. Participants will be told that, if any foods or procedures cause discomfort, they are free to refrain from eating those foods or completing those procedures without compromising total participation in the study.

### Intervention

#### Overview

The intervention is called Go for Fiber Against Risk of Diabetes (GO-FAR), which is a fully mHealth-adapted version of an existing intervention, Fiber for the Improvement of Behavior, Eating, and Risk (FIBER), which has been described elsewhere [8]. In brief, the intent of FIBER is to focus singularly on promoting greater intake of fiber via a combination of in-person group-based sessions as well as phone-based motivational interviewing and nutrition counseling, which span 3 months. GO-FAR is a fully remote mHealth adaptation that was created by mapping FIBER to its core behavior change techniques and then, ultimately, to an mHealth-compatible version [28]. In total, GO-FAR includes 3 core elements, each described below: fiber-focused messaging delivered in both video and text format up to twice per day for 3 months, biofeedback in the form of CGMs, and home-delivered high-fiber foods.

#### Intervention Messages

Video-based content was designed to target SDT and SCT constructs, including those targeted in FIBER [8]. Specifically, videos were designed to be short (<1 minute each) and cover areas such as what fiber is, where to find it, how to incorporate it into meals, how it affects blood glucose differently than other carbohydrates, how it differs in isolated or synthetic vs intrinsic forms, and how to cope with side effects that often occur (eg, digestive discomfort). Each video is delivered once per day in sequential order and after completion of the morning daily diary. Participants are encouraged to watch every video as videos build on one another in terms of content and complexity. For example, earlier videos cover basic information, such as what fiber is (a carbohydrate found in plant food). Later videos cover how fiber is different from other carbohydrates that cause increases in blood sugar, such as monosaccharides or refined starch.

In addition to the messaging videos, individual coping messages were designed based on existing evidence regarding factors that influence fiber intake, including GI responses, feeling short on time, and poor motivation for eating fiber [22]. These messages were designed using an acceptance and commitment therapy–informed approach, for example, increase in acceptance of short-term negative feelings or discomfort in pursuit of long-term goals [52]. These coping messages are delivered using a just-in-time adaptive intervention framework based on participants’ real-time EMA responses (described below). In brief, EMA responses related to key determinants of fiber intake (eg, GI discomfort, perceived time constraints, motivation, and cravings) are evaluated in real time using prespecified thresholds. When participants endorse a given factor at or above a defined threshold (eg, moderate or high levels), a corresponding coping message is delivered immediately following EMA completion and prior to video content delivery. If no factors exceed the defined thresholds, participants receive standard educational video content only. In cases in which multiple factors exceed thresholds, a prioritization schema is applied to ensure delivery of the most contextually relevant coping message. Message selection is deterministic and mapped to specific EMA responses, ensuring consistency in intervention delivery across participants.

All intervention components, including EMA prompts, coping messages, and video content, are delivered via the Insight platform and are time-stamped and logged. Engagement metrics will include EMA completion rates and intervention delivery records. These data will be used to assess intervention fidelity and participant exposure, which are critical for interpreting both feasibility and preliminary efficacy outcomes.

#### Biofeedback

Biofeedback in this study will be obtained via the use of CGMs. The CGM used will be the Dexcom G7. Participants will be given a G7 device at their baseline visit and walked through how to self-insert it with research personnel trained by the team of coauthor LJC. They will also be handed a second G7 device and instructed to self-insert it at the midpoint of the intervention (approximately 6 weeks). The CGM will not be used as a means of measuring glycemic control but, rather, will be used as a form of biofeedback to allow participants to observe the glycemic effects of adding high-fiber foods to their diet.

#### High-Fiber Foods

High-fiber foods will be delivered to participants’ homes once per week for 12 weeks. These foods are not intended to be the sole source of nutrition for participants but, rather, an additional behavior change technique (eg, adding objects to the environment) [28]. All foods have naturally occurring fiber, or intrinsic fiber. That is, they are not specialty products that have extra fiber added (isolated or synthetic), such as specialty breads, tortillas, bars, or sodas. Curated recipes corresponding to each food are included in a section of the Insight app and organized by featured ingredient. For example, one of the foods delivered is farro, and there is a corresponding “Farro” section on the Insight app that provides recipes that feature farro. Each weekly package will include one food per food group, meaning one vegetable, one fruit, one grain, and one protein (which includes nuts, beans, seeds, and lentils). Foods were chosen based on fiber content (ie, ≥3 g per standard serving) and represent all food groups except for dairy given that these foods have no fiber. Secondarily, foods were selected based on ease of procurement after the intervention (eg, cost and availability). Participants will be instructed to consume the foods as part of meals and snacks and in appropriate portion sizes.

### Data Collection

#### Phase 1

Participants will complete 2 in-person data collection sessions at the University of Oklahoma Health Campus in Tulsa, one at baseline and one after the 3-month intervention (postintervention time point). Each time, they will be asked to fast for 10 hours and have a venous blood draw done, complete anthropometric assessments, and complete a measure of capillary fasting blood glucose. Venous blood samples will be collected and processed for assessment of fasting insulin. Fasting capillary blood glucose and venous insulin will be combined to calculate the Homeostatic Model Assessment of Insulin Resistance (HOMA-IR) using established calculations (fasting glucose in mmol/L × fasting insulin in μU/mL/22.5) [53,54]. If no HbA_1c_ value is on file for the previous 3 months, HbA_1c_ will be checked using an A1CNow Self Check system (PTS Diagnostics), which will be rechecked at the postintervention time point. On 2 random days after completing the baseline visit, participants will also complete 2 separate Automated Self-Administered 24-hour dietary recalls (ASA-24) to quantify fiber intake. They will also complete surveys, including a measure of basic demographic variables. Surveys will be administered in REDCap [55,56].

During the intervention period, participants will complete daily EMAs on the Insight mHealth app maintained by the University of Oklahoma Health Campus OU Health Stephenson Cancer Center mHealth Shared Resource. This will include a run-in period, where the Insight app will deliver a morning daily diary, a midmorning diary, and a midday diary. This will decrease to only a morning daily diary during the intervention period. The morning daily diary is delivered 30 minutes after waking, before typical morning mealtimes. The midmorning and midday EMAs will be delivered approximately 2 hours after normal first and second mealtimes for the day. The morning daily diary will include a checklist-type assessment of intake of high-fiber foods and static measures such as sleep, whereas all daily diaries will assess affect, food cravings, digestive symptoms, feelings of “lack of time,” motivation to eat high-fiber foods, and urge to eat tasty food. Participants will also be presented with participant-initiated questions on the app to report when a study-provided food was eaten.

To assess feasibility of the intervention, we will evaluate the following metrics according to Bowen et al [57]: implementation, demand, acceptability, and preliminary efficacy. Implementation will be assessed via review of message delivery, which will be automatically tracked on Insight. We will also track delivery of high-fiber food packages and CGM provision as defined in the protocol. For demand, we will assess participant retention in the trial as reflected by completion of data collection activities, as well as intervention uptake. Intervention uptake will be assessed via the percentage of all delivered content viewed by participants on Insight, use of CGMs by participants for the total duration requested, and participant report of consumption of provided high-fiber foods. For acceptability, we will provide acceptability surveys at the postintervention time point that include Likert-scale questions. Finally, for preliminary efficacy, we will leverage data collection on HbA_1c_ and the HOMA-IR.

#### Phase 2

After completion of the intervention and phase 1 postintervention data collection, participants will begin an observation-only period of EMA that spans 4 weeks. During this time, they will receive no intervention but complete EMAs 3 times a day as in the run-in period. EMA variables will be the same as during phase 1. Then, after completion of this period, they will repeat 2 more random, unannounced ASA-24 measures for fiber intake (“post-observation” time point).

### Statistical Analysis

#### Power

The focus of this project is to test feasibility; thus, for phase 1, we will recruit based on existing recommendations for feasibility studies (ie, N≥30) [58]. However, we will maximize sample size for robust EMA data in phase 2. Specifically, for phase 2, the purpose is to examine factors that influence sustained intervention effects. We anticipate that all individuals who complete postintervention data collection (n=64; 32 each year, 80% retention) will begin EMAs. Per past EMA studies, we estimate 75% compliance with EMAs. For questions only asked in the morning daily diary (eg, sleep), each participant will complete approximately 21 daily diary responses (1 per day × 28 days × 0.75 compliance). Thus, with an effective sample size of 237 based on a conservatively high estimate of the correlation between daily assessment of sleep (0.7 × 1344 EMA responses), we will have 80% power to detect an association of a relatively small effect size (Cohen *f*^2^=0.034) between sleep and fiber intake. When examining associations between fiber intake and momentary predictors, which are assessed multiple times per day (eg, stress), we will be able to detect even smaller effect sizes.

#### Analytic Plan

For phase 1, we will compute descriptive statistics (for all feasibility metrics) and 2-tailed paired *t* tests to examine changes in our preliminary efficacy outcomes (fiber intake, HbA_1c_, and HOMA-IR) from baseline to the postintervention time point as a measure of preliminary efficacy. For phase 2, the primary goal is to determine whether participants maintain fiber intake after the intervention and identify real-time factors that predict adherence. Adherence will be defined as maintaining fiber intake at or above 80% [59] of postintervention levels based on ASA-24 data at the start and end of the 4-week EMA period. A logistic multilevel model will be used to predict adherence (1=maintained; 0=dropped below threshold) from EMA-measured factors (eg, affect). EMA predictors will be aggregated at the participant level (eg, mean stress across the EMA period) to assess overall exposure to these factors, whereas daily fluctuations will be modeled using within-person deviations from each participant’s mean. To assess how the influence of these predictors changes over time, we will use time-varying effect modeling, allowing for the examination of whether certain factors (eg, stress) exert stronger effects on adherence early vs late in the observation period. Additionally, we will explore the role of EMA variability, calculating within-person fluctuations (eg, SD of stress) to test whether greater instability in these factors predicts lower adherence. We will assess patterns of missing data and conduct sensitivity analyses assuming different missing data mechanisms. For example, we will consider a multiple imputation approach, which uses baseline characteristics to account for potential missing at random mechanisms [60,61].

## Results

This study was funded and initiated in July 2025. As of March 2026, the mHealth intervention content is being built, with completion of app development and trial enrollment slated to begin in April 2026.

## Discussion

### Expected Findings

This protocol describes a first-of-its-kind examination of a fully remote mHealth intervention that focuses singularly on promoting intake of fiber in young adults with prediabetes. This protocol and resulting data will shed light on two key factors: (1) the utility of an mHealth intervention in young adults and (2) the potential effects of a singular focus on fiber in prediabetes.

Diet is a central mechanism driving increased incidence of diabetes in young adults and current trends in rates of prediabetes [1,2,33]. Questions remain on the ideal diet targets to improve glycemic control and lower risk. Historically, a key focus has been on promoting a negative energy balance to facilitate weight loss, an approach that is efficacious compared to standard-of-care pharmaceutical intervention [62]. However, as in other chronic diseases, maintaining this negative energy balance is challenging. Therefore, there is interest in alternative approaches that are simple to conceptualize yet effective for improving glycemic control. From this perspective, decreasing total carbohydrates as in a ketogenic or carnivore diet is highly appealing. Carbohydrates are the nutrient responsible for increases in blood glucose; thus, their elimination would result in a decreased frequency of blood glucose spikes. Decreasing carbohydrates in this manner may also result in weight loss as individuals do succeed in losing weight on carbohydrate-restricted diets [63]. However, this may only mask underlying insulin resistance if fiber in the diet is key to improving glucose regulation. Indeed, recent evidence highlights the potential of fiber to facilitate microbiome modulation, resulting in improved glycemic control and response to pharmaceutical intervention [64]. However, this may only occur with foods high in intrinsic fiber, not isolated or synthetic fiber supplementation (eg, isolated psyllium husk) [65].

While fiber can be consumed in the context of low-carbohydrate diets, options that are both low in carbohydrate and high in intrinsic fiber are few. Furthermore, carbohydrate-containing fiber sources may be germane for reducing diabetes risk. In the empirical diabetes risk reduction diet, nuts (hazard ratio [HR] 0.57 for White adults and 0.65 for adults from minority groups) and cereal fiber (HR 0.9 for White adults and 0.95 for adults from minority groups) are both associated with reduced diabetes risk [12]. This is contrasted with red and processed meat, which has an HR of 1.34 for White adults and 1.14 for adults from minority groups [12]. Nuts are indeed low in carbohydrates, with 1.9 g of carbohydrates for every 1 g of fiber (ie, a 1.9:1 carbohydrate-to-fiber ratio) [66]. Some cereals, such as oats, have a higher carbohydrate content but still a relatively low carbohydrate-to-fiber ratio (6.7:1). Existing research shows that greater reliance on foods with less than a 10:1 ratio is associated with lower fasting glucose and HOMA-IR [66].

Cereal fiber can be consumed in isolated or synthetic forms present in specialty food products that may or may not have lower total carbohydrate content. Whether these have the same effects as intrinsic cereal fiber is not well known, and effects may be limited to cholesterol [67]. If the other food components present in intrinsic fiber sources (eg, polyphenols) are partially responsible for the protective glycemic effects, specialty food products may not prove advantageous. In their recent systematic review and meta-analysis, Colak et al [68] concluded that whole foods—as compared to supplemental sources—only improve HbA_1c_ versus other measures of glycemic control. However, most whole food–based studies included in their review appeared to be whole food sources of isolated or synthetic fibers (eg, “soy fiber in cookies”) vs food sources of intrinsic fiber (eg, soybeans). This is a vital distinction given the influx of products on the market that are markedly high in isolated or synthetic fiber.

Despite the potential benefits of eating more whole fiber, individuals may be reluctant to do so given that fiber is itself a carbohydrate and commonly coexists with digestible carbohydrates such as starch [30]. For example, per 1-cup serving, white beans contain 42.6 g of digestible carbohydrates and 13 g of fiber, whereas a food such as green beans has 4.8 g of digestible carbohydrates but only 3.5 g of fiber. Thus, if it is fiber intake—vs carbohydrates per se—that lowers risk of diabetes, a slightly higher carbohydrate intake to enable sufficient fiber may be necessary [69]. This is particularly true as fiber intake in excess of 30 g per day is associated with the greatest reduction in risk. In their systematic review and meta-analysis, Reynolds et al [69] observed a dose-response relationship between fiber intake and type 2 diabetes risk, including up to 45 g per day. Ingesting sufficient fiber via low–fiber density foods (such as green beans or nuts) may result in excessive calorie or total food intake, and fiber-dense sources (such as legumes) may be essential. The use of CGMs, as in this study, may help individuals observe the differences between acute increases in blood glucose and improved insulin sensitivity, key distinctions when adding fiber-containing foods to the diet. While the starch in foods such as beans may cause acute increases in glucose, this alone is not indicative of insulin resistance. Continuing to rely on foods such as these may improve long-term glucose regulation despite short-term increases in serum glucose levels [70].

Promoting greater intake of intrinsic fiber sources can also serve as an approach to behavior change. As outlined in the study by Ma et al [5] and others [8], by promoting greater intake of foods high in intrinsic fiber, participants are in essence merely working toward adoption of an overall healthy dietary pattern (eg, increasing intake of fruits, vegetables, whole grains, and beans or legumes) but via a singular focus on foods high in fiber. Existing data show that conventional advice to eat more plants, fruits, and vegetables or promote adoption of other disease-protective dietary patterns (eg, the Mediterranean diet) does not necessarily translate into improvements in fiber intake sufficient to meet levels that are associated with the highest disease risk reduction [71,72]. However, there is emerging evidence suggesting that greater fiber intake may be associated with greater improvements in multiple effects, such as sleep and mood, which may offer a greater return on investment, promoting and incentivizing long-term maintenance [73-75]. If intervention guidance is relatively simple and offers greater potential for perceived physiological change, it may promote intake in the long term.

### Limitations

While this study is innovative, it is not without limitations. First, as a single-arm trial, the effects of the intervention cannot be fully ascertained. Future work should include randomized trials, including comparators that feature either standard-of-care guidance (eg, DPP) or alternative diets that tap into the same psychosocial mechanisms via different dietary targets (eg, ketogenic or carnivore diet). These future trials should include more robust measures of glucose homeostasis, including oral glucose tolerance tests. Additionally, while participants are provided with food in this study, it is not a full outpatient feeding trial wherein all food is provided. This introduces variability; however, we are seeking to assess this variability in part via EMA, which could result in an intervention with greater ecological validity and potential for long-term effectiveness.

### Conclusions

The results of this study will provide pivotal evidence on the utility of a fiber-focused intervention delivered via mHealth with biofeedback and home-delivered foods to lower risk of diabetes in young adults.

## Supplementary material

10.2196/97873 Peer Review Report 1 Peer review report from Harold Hamm Foundation through the Harold Hamm Diabetes Center (HHDC) Novel Pilot Project.

## Abbreviations

ASA-24: Automated Self-Administered 24-hour dietary recall
CGM: continuous glucose monitor
DPP: Diabetes Prevention Program
EMA: ecological momentary assessment
FIBER: Fiber for the Improvement of Behavior, Eating, and Risk
GI: gastrointestinal
GO-FAR: Go for Fiber Against Risk of Diabetes
HbA_1c_: hemoglobin A_1c_
HOMA-IR: Homeostatic Model Assessment of Insulin Resistance
HR: hazard ratio
mHealth: mobile health
REDCap: Research Electronic Data Capture
SCT: social cognitive theory
SDT: self-determination theory
