# Supplementary material for: Preliminary Examination of an Innovative mHealth-Based Dietary Fiber Intervention to Improve Outcomes in Young Adults With Prediabetes: Protocol for a Single-Arm Feasibility Study
Source: JMIR Res Protoc. 2026 Sep 23;15:e97873. doi: 10.2196/97873 (PMC13600692; doi:10.2196/97873)
Supplement: Supplementary file 1 [file resprot-v15-e97873-s001.pdf]

Harold Hamm Diabetes Center
Summary of Review

Principal Investigator: Ashlea C Braun

Funding Mechanism: Novel Pilot Grant

Title: Preliminary Examination of an Innovative mHealth-Based Dietary Fiber Intervention to Improve Outcomes in Young Adults with Prediabetes: A Feasibility Study

Funding Decision: Recommended for funding

Summary Statement:
The overall objective of this study is to establish a preliminarily framework to test a scalable mHealth-based intervention to improve dietary fiber intake and diabetes risk among young adults with prediabetes using: SA1) a direct home-delivery of high dietary fiber foods and biofeedback using continuous glucose monitors and SA 2) factors that determine sustained use after study ends. Combining mHealth, direct food delivery, and biofeedback for diabetes prevention in young adults was considered somewhat novel. Some reviewers and committee members viewed a single dietary focus on fiber as a positive, with a condensed message that is easily understood, while others viewed it too narrow with a suggestion of a slight shift in the narrative towards precision nutrition vs. negative perceptions of carbohydrates. The potential for future federal funding is considered high, which was an important aspect considered in the funding decision for this project.

There was some concern with the budget, as approximately half of the funds going to the PI and a GRA (with no responsibilities attributed to PI).

Harold Hamm Diabetes Center
Novel Pilot Project Grant Review Form

Principal Investigator of Grant Proposal: Ashlea Braun

Title: Preliminary Examination of an Innovative mHealth-Based Dietary Fiber Intervention to Improve Outcomes in Young Adults with Prediabetes: A Feasibility Study

Please use the following NIH Grant scoring criteria when assigning scores, from 1 (highest) to 9 (lowest). Start with a score of 5 and go up or down accordingly. Please use the complete scoring range:

Degree of Impact	Impact Score	Descriptor	Additional Guidance on Strengths/ Weaknesses
High	1	Exceptional	Exceptionally strong with essentially no weaknesses
	2	Outstanding	Extremely strong with negligible weaknesses
	3	Excellent	Very strong with only minor weaknesses
Moderate	4	Very Good	Strong but with numerous minor weaknesses
	5	Good	Strong but with at least one moderate weakness
	6	Satisfactory	Some strengths but also some moderate weaknesses
Low	7	Fair	Some strengths but with at least one major weakness
	8	Marginal	A few strengths and a few major weaknesses
	9	Poor	Very few strengths and numerous major weaknesses

Definitions:
Minor: easily addressable weakness that does not substantially lessen the impact of the project.
Moderate: weakness that lessens the impact of the project.
Major: weakness that severely limits the impact of the project.

Use as much space as you need to address the criteria listed below:

1. Innovation and Scientific Merit: Is the work novel and is it likely to open new opportunities for future work? Does it still show potential to advance research or practice in the fields relevant to the project? Is the work relevant to clinical and translational research or community-based practice?
Score: 4

The strengths are distilling the principles of the Diabetes Prevention Program down into a single take-away message (eat more fiber), which could improve adherence yet still provide a clinically meaningful intervention, and utilizing mHealth to conduct ecological momentary assessments to deliver messaging and give feedback to participants in real time. Food delivery also adds an intervention desired by participants in a preliminary study, clearly building on the previous work to design an intervention more acceptable to participants. The aims target reduction in diabetes risk, which is clearly relevant to the population.

The strengths—and the focus on diabetes prevention—get a little lost in the background/significance and preliminary studies sections. The section on negative perceptions of carbohydrate-containing foods doesn't seem to fit, and although an analysis is in progress, this data seems borderline anecdotal and

fails to demonstrate that carbohydrate-containing foods are demonized in this population. This distracts from the overall narrative of the proposal and its desired outcomes.

Delivery of educational content via mHealth is currently desired by participants in the FIBER trial and does represent an area of interest for extramural funders. The FIBER trial has shown that the educational curriculum there (in-person classes plus one-on-one phone visits with—I assume—a registered dietitian) has been successful in producing behavior change. It is not clear if this same curriculum is what will be translated to the mHealth intervention, and no pilot testing of the mHealth delivery seems to have been done.

2. Soundness of the Approach and Research Design: Are the methods proposed reasonable and likely to succeed? **Score**: 4

Fiber intake is a sound approach to help reduce the messaging from the Diabetes Prevention Program to a key point, which may be more manageable for participants. There are good indications from the ongoing FIBER trial that this message is acceptable to participants and can have clinically meaningful effects.

The use of CGMs is questionable to provide feedback to participants on their fiber intake, particularly if we go with the assumption that the average participant has a negative perception of high carbohydrate foods. High fiber foods contain carbohydrates, and the average user would likely not be able to reason that blood glucose levels rose after consuming a high-fiber food but that the increase was less than it would have been after consuming an identical meal with lower fiber. (Would a participant compare their glucose after a high fiber meal to a meal of the same size, at the same time of day, under the same conditions, but with lower fiber? Would participants know how much of a rise in glucose is acceptable or "good" after a meal? If we go with the idea that they think all carbohydrates are bad and maintaining a steady low blood glucose is good, any food containing carbohydrate would elicit "negative" feedback on a CGM, which may lead to reduced intake of high fiber foods.) If the research team wants to collect this data for exploratory data, that approach would be more straightforward, but to use it as biofeedback to motivate participants is unlikely to be effective.

With the discussion of individual autonomy, allowing participants to choose their own high fiber foods for delivery would be more in keeping with self-determination theory. Even with inclusion of recipes with high-fiber food delivery, participants will likely need to supplement with their own foods to create these recipes and need adequate cooking facilities, time, and skills, compared to delivering meals or snacks that need little to no preparation and would be more likely to be consumed.

The FIBER trial is the strongest argument that fiber intake can be increased in this population. What I'm not seeing is clear indication that the intervention in the FIBER trial (in-person education with phone-based counseling) will translate to an mHealth delivery in the current proposal. 12 weeks of intervention (with half including support in a group setting and half in one-on-one conversations) to be replaced by electronic messaging? I'm skeptical that significant behavior change could be expected, especially if significant numbers of participants do have a negative perception of carbohydrates. Ideally, some—even small—pilot testing of the intervention delivered via mHealth would strengthen the proposal.

3. Qualifications and Experience of the Investigator(s): Does the PI have a background in the proposed area of research? Is the PI experienced in relevant scientific techniques and proposed experimental design? Do Co-Is provide relevant experience that strengthens the objectives of the proposal? **Score**: 3

The FIBER trial is the most compelling section to convince reviewers that Dr. Braun can achieve results in this project—the FIBER trial has shown positive results and participant adherence, with a strong methodology combining quantitative and qualitative measures.

The group has not published together, which is acknowledged in the proposal. Co-Is mostly seem to provide resources or technical assistance rather than complete collaboration. Dr. Hébert has expertise in EMAs, but have they been used to deliver intervention in previous studies, or have they been data collection methods? If they have been used to deliver significant amounts of intervention or education, please specify more clearly. As is, this reads that Dr. Hébert may have used EMAs but perhaps not as the main method of intervention, which seems a significant change in their use. Ideally, I would like to see some pilot testing translating the FIBER curriculum to an mHealth delivery with both Drs. Braun and Hébert involved.

4. First-year milestones: Are the first-year milestones likely to be met? **Score:** 2
Target recruitment seems reasonable, based on the success of the FIBER trial, and since the project would be replicated if funded in the second year, results should be available to guide further funding.

5. External Funding Opportunities: Does the project demonstrate potential for extramural funding? Is there a realistic expectation that Federal funding will be attainable by the end of the second year of the pilot grant? **Score**: 7
Even with successful results, trialing feasibility in this grant to an R01 seems unlikely to succeed; foundation funding (i.e., ADA as indicated) may be more realistic.

6. Budget: Is the budget realistic for the work being proposed? **Score: 5**
Bulk of the budget is salary for PI and a GRA—with nearly half the budget dedicated to the GRA, there are no responsibilities described in the budget description.

7. Additional comments, if any:

8. **Indicate your overall score.** _____4_________

Harold Hamm Diabetes Center
Novel Pilot Project Grant Review Form

Principal Investigator of Grant Proposal: Ashlea C Braun

Title: Preliminary Examination of an Innovative mHealth-Based Dietary Fiber Intervention to Improve Outcomes in Young Adults with Prediabetes: A Feasibility Study

Please use the following NIH Grant scoring criteria when assigning scores, from 1 (highest) to 9 (lowest). Start with a score of 5 and go up or down accordingly. Please use the complete scoring range:

Degree of Impact	Impact Score	Descriptor	Additional Guidance on Strengths/ Weaknesses
High	1	Exceptional	Exceptionally strong with essentially no weaknesses
	2	Outstanding	Extremely strong with negligible weaknesses
	3	Excellent	Very strong with only minor weaknesses
Moderate	4	Very Good	Strong but with numerous minor weaknesses
	5	Good	Strong but with at least one moderate weakness
	6	Satisfactory	Some strengths but also some moderate weaknesses
Low	7	Fair	Some strengths but with at least one major weakness
	8	Marginal	A few strengths and a few major weaknesses
	9	Poor	Very few strengths and numerous major weaknesses

Definitions:
Minor: easily addressable weakness that does not substantially lessen the impact of the project.
Moderate: weakness that lessens the impact of the project.
Major: weakness that severely limits the impact of the project.

Use as much space as you need to address the criteria listed below:

1. Innovation and Scientific Merit: Is the work novel and is it likely to open new opportunities for future work? Does it still show potential to advance research or practice in the fields relevant to the project? Is the work relevant to clinical and translational research or community-based practice? **Score:** 3

The nutrition field is in profound need for highly rigorous mHealth interventions aimed at impacting a dietary change goal. Diabetes remains a prominent, chronic health conditions to be addressed. The data on fiber *per se* as an established effective intervention is unclear, but is being outshined by alternative/modern dietary approaches e.g., a low-carbohydrate diet (both in the clinical research realm and public perception). Highly innovative and scientifically necessary nutritional studies have transitioned towards multi-diet interventional approaches, while this study remains a single-diet approach.

2. Soundness of the Approach and Research Design: Are the methods proposed reasonable and likely to succeed? **Score**: 3

Inherent issues: 1) no control group, 2) single diet/single nutrient intervention, 3) recruitment capabilities not well evidenced for aggressive timeline proposed

3. Qualifications and Experience of the Investigator(s): Does the PI have a background in the proposed area of research? Is the PI experienced in relevant scientific techniques and proposed experimental design? Do Co-Is provide relevant experience that strengthens the objectives of the proposal? **Score**: 1

PI has direct experience in the primary facets of this research: fiber intake, dietary behavior change methods, mHealth strategies. Co-investigators and other study team members are well-positioned to conduct this research based on their funding history, research experience and dedicated time to commit to the study. Appropriate personnel are dedicated to provide statistical support and to implement the proposed research.

4. First-year milestones: Are the first-year milestones likely to be met? **Score:** 2

Milestones appear too aggressive, especially regarding recruitment of a strict population. Additional support evidencing the ability to recruit this study population in a tight time-frame and additional funds dedicated to marketing/recruiting would improve confidence in the timeline e.g., submitting findings for publication in month 7 is unlikely given a >5 month intervention length (enrollment to final assessment).

5. External Funding Opportunities: Does the project demonstrate potential for extramural funding? Is there a realistic expectation that Federal funding will be attainable by the end of the second year of the pilot grant? **Score**: 3

Funding for single-nutrient interventions (e.g., fiber) may not be as enthusiastically received as the mHealth concept or even commercialization potential (SBIR/STTR vs R01). Additionally, shifting the overall narrative towards precision nutrition (e.g., nutrition for precision health trial) may be more enticing for federal funding sources as opposed to “Negative perceptions of carbohydrate foods.” The latter is minimally evidenced, has (to date) null clinical impact and in the context of growing evidence of alternative approaches such as low-carbohydrate diets to improve glycemic control, may be limited in funding scope from a federal perspective.

It’s difficult to understand the co-existing grant narratives that minimizes the positive impact of both the DPP and low-carbohydrate approaches while suggesting a fiber-only approach (a single-nutrient part of DPP) to be the potential best option for glycemic control. A transition towards modern multi-diet/precision nutrition methods may help alleviate this disconnect.

6. Budget: Is the budget realistic for the work being proposed? **Score: 1**

Appropriate

7. Additional comments, if any:

Excellent application, mHealth approaches with nutrition-based outcomes are a key need in the field. Minor weaknesses are present as described throughout. Few larger concerns, such as the methods and low-potential for a major clinical impact of the intervention dampen enthusiasm. Still, the completion of

this project will 1) add important information to the field and 2) provide critical data for future federal funding opportunities.

8. **Indicate your overall score.** _______3_______

Harold Hamm Diabetes Center
Novel Pilot Project Grant Review Form

Principal Investigator of Grant Proposal: Ashlea C Braun

Title: Preliminary Examination of an Innovative mHealth-Based Dietary Fiber Intervention to Improve Outcomes in Young Adults with Prediabetes: A Feasibility Study

Please use the following NIH Grant scoring criteria when assigning scores, from 1 (highest) to 9 (lowest). Start with a score of 5 and go up or down accordingly. Please use the complete scoring range:

Degree of Impact	Impact Score	Descriptor	Additional Guidance on Strengths/ Weaknesses
High	1	Exceptional	Exceptionally strong with essentially no weaknesses
	2	Outstanding	Extremely strong with negligible weaknesses
	3	Excellent	Very strong with only minor weaknesses
Moderate	4	Very Good	Strong but with numerous minor weaknesses
	5	Good	Strong but with at least one moderate weakness
	6	Satisfactory	Some strengths but also some moderate weaknesses
Low	7	Fair	Some strengths but with at least one major weakness
	8	Marginal	A few strengths and a few major weaknesses
	9	Poor	Very few strengths and numerous major weaknesses

Definitions:
Minor: easily addressable weakness that does not substantially lessen the impact of the project.
Moderate: weakness that lessens the impact of the project.
Major: weakness that severely limits the impact of the project.

Use as much space as you need to address the criteria listed below:

1. Innovation and Scientific Merit: Is the work novel and is it likely to open new opportunities for future work? Does it still show potential to advance research or practice in the fields relevant to the project? Is the work relevant to clinical and translational research or community-based practice?
Score: 2

The project is highly innovative, combining mHealth, direct food delivery, and biofeedback for diabetes prevention in young adults. It addresses a critical gap in current interventions and has potential for significant impact.

2. Soundness of the Approach and Research Design: Are the methods proposed reasonable and likely to succeed? **Score**: 3

The two-phase design is well-thought-out, with appropriate measures and a focus on both immediate and sustained behavior change. The sample size is adequate for a pilot study.

3. Qualifications and Experience of the Investigator(s): Does the PI have a background in the proposed area of research? Is the PI experienced in relevant scientific techniques and proposed experimental design? Do Co-Is provide relevant experience that strengthens the objectives of the proposal? **Score**: 2

The PI and Co-I have relevant expertise in mHealth interventions, dietary research, and health outcomes studies, making them well-qualified to conduct this research.

4. First-year milestones: Are the first-year milestones likely to be met? **Score:** 3

The proposed milestones are realistic and achievable within the first year, covering intervention development, participant recruitment, and preliminary data analysis.

5. External Funding Opportunities: Does the project demonstrate potential for extramural funding? Is there a realistic expectation that Federal funding will be attainable by the end of the second year of the pilot grant? **Score**: 4

The project shows strong potential for attracting extramural funding, particularly from NIH, given its focus on diabetes prevention and innovative approach.

6. Budget: Is the budget realistic for the work being proposed? **Score:** 3

The budget appears to be carefully considered, with detailed breakdowns for personnel, supplies, and other costs. The researchers have made efforts to keep the project within budget constraints while still maintaining scientific integrity. They have modified their measure of insulin resistance from the Triglyceride-Glucose (TyG) index to HOMA-IR in response to reviewer comments. This change increased costs, but Dr. Braun will use internal start-up funds ($5,760) to cover the measurement of fasting insulin and glucose levels for HOMA-IR evaluation. The willingness to use internal funds to support the project demonstrates commitment and resourcefulness, which is a positive aspect for the review.

7. Additional comments, if any:

8. **Indicate your overall score.** _____2_________
